# An investigation of intensity-modulated radiation therapy versus conventional two-dimensional and 3D-conformal radiation therapy for early stage larynx cancer

**DOI:** 10.1186/1748-717X-5-74

**Published:** 2010-08-26

**Authors:** Daniel Gomez, Oren Cahlon, James Mechalakos, Nancy Lee

**Affiliations:** 1 Department of Radiation Oncology, Memorial Sloan-Kettering Cancer Center, New York, NY, USA; 2 Department of Medical Physics, Memorial Sloan-Kettering Cancer Center, New York, NY, USA

## Abstract

**Introduction:**

Intensity modulated radiation therapy (IMRT) has been incorporated at several institutions for early stage laryngeal cancer (T1/T2N0M0), but its utility is controversial.

**Methods:**

In three representative patients, multiple plans were generated: 1) Conventional 2D planning, with the posterior border placed at either the anterior aspect ("tight" plan) or the mid-vertebral body ("loose" plan), 2) 3D planning, utilizing both 1.0 and 0.5 cm margins for the planning target volume (PTV), and 3) IMRT planning, utilizing the same margins as the 3D plans. A dosimetric comparison was performed for the target volume, spinal cord, arytenoids, and carotid arteries. The prescription dose was 6300 cGy (225 cGy fractions), and the 3D and IMRT plans were normalized to this dose.

**Results:**

For PTV margins of 1.0 cm and 0.5 cm, the D95 of the 2D tight/loose plans were 3781/5437 cGy and 5372/5869 cGy, respectively (IMRT/3D plans both 6300 cGy). With a PTV margin of 1.0 cm, the mean carotid artery dose was 2483/5671/5777/4049 cGy in the 2D tight, 2D loose, 3D, and IMRT plans, respectively. When the PTV was reduced to 0.5 cm, the the mean carotid artery dose was 2483/5671/6466/2577 cGy to the above four plans, respectively. The arytenoid doses were similar between the four plans, and spinal cord doses were well below tolerance.

**Conclusions:**

IMRT provides a more ideal dose distribution compared to 2D treatment and 3D planning in regards to mean carotid dose. We therefore recommend IMRT in select cases when the treating physician is confident with the GTV.

## Introduction

Larynx cancer is the most common head and neck malignancy in the United States and approximately half of these malignancies present at an early stage (T1-T2N0). Treatment of this early disease is controversial because there are several effective treatment modalities including radiation therapy, endoscopic resection and open partial laryngectomy. No single modality has been proven to be superior to the others [1]. The goal of any therapy is cure with larynx preservation, high voice quality, and minimal morbidity. Although endoscopic resection has gained popularity over the past decade, many still consider definitive radiation to be the mainstay of therapy.

Many institutions, including our own, have recently incorporated intensity modulated radiation therapy (IMRT) into the treatment of early stage glottic cancer for selected patients. IMRT has the capability of producing highly conformal dose distributions with steep dose gradients to target areas of concern while sparing nearby critical organs in the neck. In addition, IMRT may produce better target coverage, leading to improved local control. However, a recent editorial questioned the role of IMRT in treating early larynx cancer and highlighted potential pitfalls of using IMRT in this scenario [2].

The following study provides a dosimetric comparison between IMRT, conventional techniques, and 3D planning for the treatment of early glottic cancer. We aim to show that, at least in select cases such as bulkier lesions or in patients with short, thick necks, IMRT can improve target coverage while simultaneously minimizing the dose to sensitive structures in the neck. By doing so, IMRT may be able to further improve local control while minimizing toxicity for these patients.

## Materials and methods

Three representative patients treated with definitive radiation using IMRT at Memorial Sloan-Kettering Cancer Center in the last year were selected for a treatment planning study. Criteria for inclusion were T1N0 or T2N0 squamous cell carcinoma of the larynx. Two of the patients selected had T1N0 tumors and one patient had a bulky T2N0 tumor. Patients were staged with direct laryngoscopy, computed tomography (CT) scan, and positron emission tomography (PET) scans. Patients were treated to the larynx without elective nodal irradiation.

Patients were immobilized in the supine position with a 5-point thermoplastic mask. Treatment planning CT scans were obtained from the top of the skull to the lower part of the neck with a 3-mm slice thickness. Intravenous contrast was used in two patients; one patient did not receive it due to an iodine allergy. Three different treatment plans were generated for each patient: 1) 2D opposed laterals (single slice) assuming a larynx contour and no CT, 2) 3D planning, using the entire larynx as the clinical target volume (CTV), and 3) IMRT, utilizing the same definition as 3D planning for the CTV. The 3D and 2D plans utilized the same beam configuration, but the conformal plan used 3D information to design apertures and normalize the plan. For anteriorly located lesions, the plans include a centrally placed 0.5 cm bolus on the skin over the treatment field. The dose to the planning target volume (PTV) in all patients was 6300 cGy in 225 cGy fractions, over a course of 38 days. This dose schedule corresponds to a nominal standard dose, which is used to compare the effect of different dose regimens, of 1905 ret, the unit for nominal standard dose.

### 2D Opposed laterals

To simulate the case in which a single slice plan is developed from a larynx contour alone, a generic larynx contour was drawn according to department guidelines for these cases: 1 cm from the anterior skin surface, and consisting of two lobes at 150 and 210 degrees from the vertical, each lobe being 1.8 cm in length for males or 2.2 cm in length for females. Right and left lateral treatment fields were created using a number of different wedge angles. The collimator angle was chosen such that the posterior jaw of the lateral fields was parallel to the cervical spine. In an attempt to cover the clinical range of 2D larynx treatments among different institutions, two different plans per patient were created: one in which the posterior edge of the fields coincided with the anterior surface of the vertebral bodies (referred to as the *tight clinical plan*), and one in which the posterior edge was in the middle of the vertebral body (labeled the *loose clinical plan*). Dose was calculated without inhomogeneity corrections and a dose of 100% was assigned to the isocenter. The isodose line which covered the larynx and had a reasonably straight posterior edge, at the discretion of the authors, was chosen as the prescription isodose. The wedge angle chosen for the plan was the one which concentrated a dose of 102-105% anteriorly. Only the isocenter slice was used for plan evaluation. Once an acceptable plan was obtained, inhomogeneity calculations were turned on and the plan was recalculated for comparison to the other plans.

### 3D planning/IMRT

Relevant structures were manually contoured on each axial CT scan slice by a head and neck radiation oncologist. The gross tumor volume was defined as the bilateral true vocal cords, to include any gross disease that can be delineated by the treating radiation oncologist (though it is often difficult to determine the region of gross disease on imaging except in the case of bulkier lesions). The CTV for each plan consisted of the larynx (false and true vocal cords, anterior and posterior commissure, arytenoids and aryepiglottic folds) as well as the subglottic region, extending from the level of the hyoid bone superiorly to the bottom of the cricoid cartilage inferiorly. Two PTV volumes were generated with varying margins from the clinical target volume, 1.0 cm and 0.5 cm. A 1.0 cm margin is generally used to ensure adequate coverage when there is greater uncertainty as to patient setup. The 3D plans consisted of two fields, with a beam configuration identical to the 2D plans, and the IMRT plans consisted of 3-4 anterior fields. To ensure that these plans were consistent with the 2D plans, we visually verified that the PTV coverage superiorly was set below the level of the hyoid bone and extended inferiorly to the level of the cricoids. The entire spinal cord, the bilateral carotid arteries, and the bilateral arytenoids were contoured as organs at risk (OAR). The 3D plans were normalized such that the PTV D95 was equal to the prescription dose. The IMRT plans were created for each PTV and also normalized such that the PTV D95 was equal to the prescription dose and the maximum PTV dose was 105% or less. Optimization was performed by lowering the desired mean dose to the carotid artery so that it did not exceed 105% of the prescription dose (PTV D95). Thus, contouring of the carotid arteries was critical in generating the plan.

The beam arrangement for the 3D and IMRT plans was the same as those used for treatment. However, for consistency, the plans were re-optimized to all conform to the same constraints. All plans had four anterior oblique beams, two on each side. Since the primary purpose of this exercise was to compare conventional techniques for treating the larynx with 3D and IMRT, we did not test different beam arrangements for the conformal plans.

As an adjunct to the above analysis, we generated a second plan of one of the three patients who had T1N0, anteriorly-located disease, based on the premise that these tumors often involve the anterior third of the true vocal cord and thus sparing the arytenoids would in turn reduce carotid dose. We reported this plan as *IMRT no arytenoids*.

### Plan evaluation

Plans were compared based on the following criteria: CTV and PTV coverage as indicated by D95 and D90, maximum PTV dose (Dmax), mean carotid artery dose, mean arytenoid dose, and maximum spinal cord dose.

## Results

**Figure 1:**
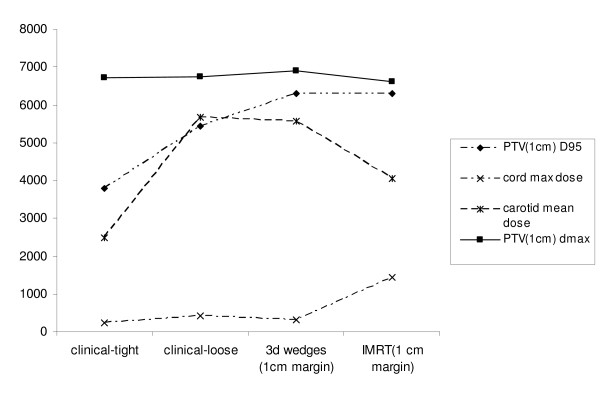
**Dosimetric Characteristics of Treatment Plans, One Centimeter PTV Margin (Dose in cGy on the Y-axis)**.

Table 1a depicts tumor coverage averaged over all three patients with a 1.0 cm margin in the tight clinical 2D, loose clinical 2D, and 3D/IMRT plans. The D95 ranged from 3781 cGy in the tight clinical 2D plan (60% of the prescription dose) to 6300 cGy in the IMRT and 3D plans (100% of the prescription dose). The hot spot, as measured by Dmax, was comparable in the four plans but was highest in the 3D plan, at 6913 cGy. Table 2a demonstrates normal structure parameters of the four different plans. The mean carotid dose was lowest in the 2D tight clinical plan, at 2483 cGy, and second lowest in the IMRT plan, at 4049 cGy. The spinal cord doses were all well below tolerance, with the maximum spinal cord dose of 1437 cGy in the IMRT plan. The arytenoid doses were comparable in all four plans, and ranged from 6289 - 6500 cGy. Figure 1 is a graphical representation of key comparisons in Tables 1a and 2a.

**Table 1:** Target Structure Dosing Comparison (PTV margin in parentheses)

	Dmax	CTV D90	PTV D90	CTV D95	PTV D95
**A**					

2D Tight (1 cm)	6713	6104	5782	5970	3781

2D Loose (1 cm)	6736	6233	6045	6111	5437

3D CRT (1 cm)	6913	6479	6437	6424	6300

IMRT (1 cm)	6610	6467	6431	6437	6300

**B**					

2D Tight (5 mm)	6685	6104	5782	5970	5372

2D Loose (5 mm)	6711	6233	6045	6111	5869

3D CRT (5 mm)	6806	6439	6367	6404	6300

IMRT (5 mm)	6615	6428	6367	6400	6300

**Table 2:** Normal Structure Dosimetric comparison of Radiation Plans

	A) 1 cm margin			B) 0.5 cm margin		
	**Arytenoid Mean (cGy)**	**Carotid Mean (cGy)**	**Spinal Cord Dmax (cGy)**	**Arytenoid Mean (cGy)**	**Carotid Mean (cGy)**	**Spinal Cord Dmax (cGy)**

2D-Tight	6289	2483	228	6289	2483	228

2D-Loose	6351	5671	407	6351	5671	407

3D wedges	6500	5777	374	6466	4371	251

IMRT	6500	4049	1437	6470	2577	1482

**Figure 2:**
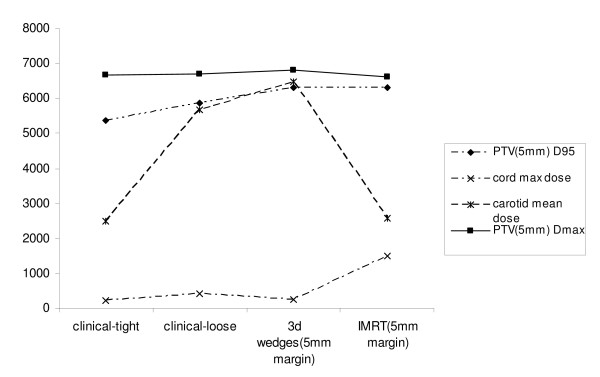
**Dosimetric Characteristics of Treatment Plans, 0.5 cm PTV Margin (Dose in cGy on Y-axis)**.

In the next step of analysis, we reduced the PTV margin from 1.0 cm to 0.5 cm, as there is no consensus regarding the appropriate expansion that should be utilized in this disease. Target structure comparisons are given in Table 1b. The margin contraction improved the D95 of the tight and loose clinical plans, now 5372 cGy and 5869 cGy, respectively. However, the D95 was still greatest in the IMRT and 3D plans, where it was optimized to 6300 cGy. Table 2b depicts normal structure doses between the four plans. While the carotid mean dose was significantly decreased in the IMRT plan by reducing the margin size, from 4049 cGy to 2577 cGy, as expected, the normal structure mean doses were unchanged in the 2D plans, where the anterior and posterior borders, and thus the dosimetry to normal structures, were independent of the size of the PTV expansion. In a comparison between the IMRT and 3D plans, the mean carotid artery dose remained substantially better with IMRT (2577 cGy compared to 4371 cGy with 3D planning). The relative doses of the arytenoids and the spinal cord did not change by altering the margin. Figure 2 demonstrates target and normal structure dosing with a 0.5 cm margin in graphical form.

**Figure 3:**
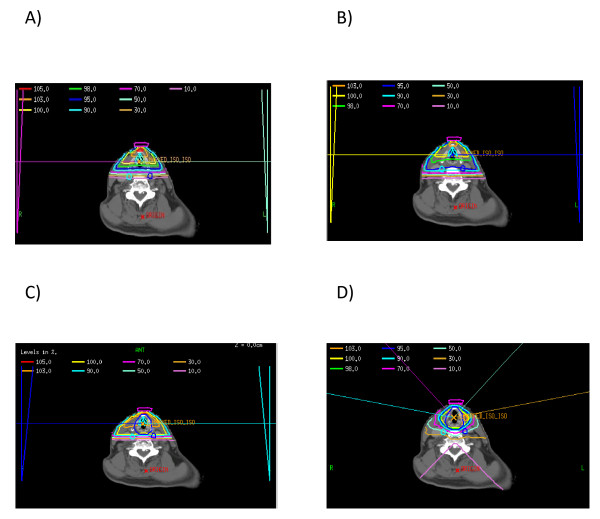
**Representative Axial Slices of Four Different Plans, a) tight clinical 2D plan, b) loose clinical 2D plan, c) 3D plan with 0.5 cm expansion from CTV to PTV, and d) IMRT plan with 0.5 cm expansion from CTV to PTV**. The PTV is delineated in Figures 3c and 3d by the dark blue thick line encompassing the larynx.

Figure 3 demonstrates axial slices from four different plans: a) the tight clinical 2D plan, b) the loose clinical 2D plan, c) 3D plan, 0.5 cm margin and d) IMRT plan, 0.5 cm margin. This figure shows that the loose clinical plan provides better target coverage than the tight clinical plan, and similar coverage to the two conformal plans, with the tradeoff of increased dose to the carotid artery. Furthermore, because no optimization was performed for the PTV in the 2D clinical plans, any relationship between the coverage of the PTV and the level of expansion was dependent on the spatial relationship between the expanded borders of the PTV and the pre-determined boundaries of the clinical plan. Thus, it is clear when examining the axial slices on Figures 3a and 3b that as the PTV expansion decreases in size (from 1.0 cm to 0.5 cm), the percentage of target volume contained within the pre-defined borders of the 2D clinical plans increases. This increased coverage is the reason why the PTV D95 and Dmax increase with both 2D clinical plans as the CTV to PTV margin decreases.

When comparing the two conformal plans, we show that both IMRT and 3D planning provide excellent coverage to the target volume, and are optimized as such. However, utilizing various beam arrangements and inverse planning, the IMRT plan provides more conformality in regards to the carotid arteries. As noted above, the arytenoids receive similar doses, and the spinal cord receives very low doses in both plans.

Table 3 compares the dose distributions to the carotid arteries in a patient with T1N0 glottic cancer and an anterior lesion. The 3D and IMRT plans were compared with a 0.5 cm margin, as these would give the lowest dose to the carotid arteries and thus provide the most conservative estimate of the advantages of sparing the arytenoid cartilage in selected patient. The table demonstrates that sparing the arytenoids provides a substantial benefit (greater than 50% in mean carotid dose) compared to any other plan.

**Table 3:** Comparison of carotid artery doses in T1N0 patient with anterior lesion and arytenoid sparing (Prescription dose 6300 cGy).

	Bilateral Carotid Dmean (cGy)	Bilateral Carotid Dmax (cGy)
Clinical Tight Plan	1493	5295

Clinical Loose Plan	5363	6302

3D Plan (0.5 cm margin)	3417	6365

IMRT plan (0.5 cm margin)	1946	5403

IMRT plan with arytenoid sparing	804	3032

## Discussion

Early stage glottic cancer is a highly curable malignancy which can be treated with either larynx sparing surgery (laser excision, cordectomy, or hemilaryngectomy) or radiation [1]. Because there is not a randomized trial to guide treatment decisions, the management of this disease remains controversial. However, at most institutions, radiotherapy is still considered the mainstay of treatment. Because both treatment modalities offer similar rates of cure, decisions regarding which therapy to pursue often lie on the anticipated toxicity profile of a particular regimen. Other factors such as tumor location and extent of disease, co-morbid illnesses and physician and patient preference also impact the final treatment decision. The ultimate goal of any therapy is cure, larynx preservation, high voice quality and overall high quality of life.

Radiation therapy has typically been delivered using a pair of lateral opposed, low energy photon fields that encompass the entire larynx (cobalt to 6 MV) as seen in Figure 3a and 3b. Typical field sizes range from 5 × 5 cm to 6 × 6 cm. Fifteen or 30-degree wedges are often used and improve the dose homogeneity throughout the vocal cords, especially for mid and posterior tumors. The superior and inferior borders are traditionally placed at the top of the thyroid cartilage and bottom of the cricoid cartilage, respectively. Anteriorly, a 1 cm flash with bolus is used. Posteriorly, the field edge is usually placed between the anterior edge of the vertebral body and the middle of the vertebral body. This treatment has consistently produced excellent outcomes with local control rates of 90-95% for T1 lesions and 75-80% for T2 lesions.

Given the excellent results with conventional treatment, some have been reluctant to change technique. In a recent editorial, Feigenberg et al thoughtfully outlined why IMRT offers little benefit and may in fact be of detriment [2]. The authors outline several key arguments in their paper which we will address in our Discussion. First, how can IMRT or any other form of conformal radiation improve upon the excellent rates of local control already achieved with conventional techniques? Second, will the routine use of IMRT lead to a higher risk of marginal failures and lower rates of local control due to smaller planning target volumes? In addition, will IMRT underdose the skin and anterior commissure due to limitations in dose-calculating algorithms, resulting in more local failures? Finally, can IMRT further reduce the risk of major morbidity from the already low rate?

We have shown in this paper that, if a physician is confident in the appropriate PTV to be used for treatment planning, IMRT results in better target coverage than conventional planning. In a tight clinical plan with the posterior border placed at the anterior edge of the vertebral body, PTV coverage is compromised. In this study, the tight clinical plans resulted in a D95 of 60% of prescription and loose clinical plans resulted in a D95 of 86% of prescription to the PTV. In contrast, the IMRT plans were optimized to a D95 of 100%. Also, in the superior-inferior direction, standard field sizes can lead to tumor under-dosing, particularly for bulky T2 lesions with significant supra- or sub-glottic extension. It is well documented that the local control rate after definitive radiation is considerably lower for T2 tumors than T1 tumors. This, in part at least, results from inadequate target coverage for bulkier lesions, particularly since a standard expansion from 5 × 5 cm (T1 tumors) to 6 × 6 cm (T2 tumors) is used with no alteration of the posterior border. This "fixed" increase in field size almost certainly does not adequately account for the differences in the extent of tumor in all cases. Finally, it is evident that regardless of the PTV expansion, the Dmax, and thus the magnitude of the hot spot was less with the IMRT plans. Indeed, when using a clinical wedge plan, the hot spots can approach 12%, which could also compromise long-term vocal function.

Another situation in which IMRT may hold advantages is in patients with thick, short necks. Because of the difficulty with hyperextension, lateral beams cannot cover the inferior aspect of the field due to shoulder obstruction. In these cases, anterior oblique beams are usually used to cover the inferior extent of the target volume. This leads to increased dose to the lung apices. This strategy was indeed utilized on one of the patients in this analysis. In addition to this study, other investigators have examined techniques to maximize the therapeutic ratio in the case of shoulder obstruction. For example, Yom et al. reported outcomes using a "caudal tilt" technique in the postlaryngectomy or pharyngectomy setting. The technique involves the angling of noncoplanar beams in the caudal direction while using 3D planning to deliver dose inferior to the standard three-field match line. The authors reported high 2-year locoregional control rates while shielding a larger amount of posterior lung as compared to the standard 3-field technique [3]. These same principles are used when altering beam angles in the IMRT setting.

With proper target delineation and adequate margins, IMRT should not lead to higher rates of marginal failures and may improve upon the already high rates of local control. The concern about marginal failures was present when IMRT was introduced into routine practice for each tumor site. However, there is no evidence that IMRT leads to higher rates of marginal failures in any disease site [4-6]. On the contrary, IMRT seems to have increased tumor control in both prostate and head and neck tumors by allowing for dose escalation and better target coverage. A number of papers in the past have shown that there is a dose response relationship for larynx cancer, particularly in terms of utilizing a higher dose per fraction [7-9]; thus, proper coverage of the target is critical for definitive radiotherapy in order to maximize local control and minimize patients who will need a laryngectomy.

As a second analysis of this study, we compared IMRT with a 3D conformal technique. Indeed, many of the issues pertaining to conventional techniques, such as dose tradeoff between target structures and normal tissue, and the need for larger margin volumes, would be addressed by the latter technique, in which normal structures could be specified and constraints set to achieve dose escalation. Indeed, while caution should be used and individualized based on the physician's comfort level, CTV to PTV margins as low as 0.3 cm have been utilized with conformal techniques.

We found in this study that, while IMRT and 3D conformal techniques were similar in terms of target coverage and the "clinically meaningful" dose to normal structures (the Dmax to the spinal cord was well below tolerance in all techniques), IMRT demonstrated a significant improvement in terms of the dose to the carotid arteries. For example, a common belief is that, when CT based planning is utilized, an appropriate CTV to PTV margin is 0.5 cm. Even at these relatively tight margins, the mean dose to the carotid arteries was almost 2000 cGy lower when utilizing IMRT than the 3D plan. This dose was lowered even further when an arytenoid sparing plan was utilized, in the case of a patient with a T1N0 lesion located anteriorly.

There is sufficient data that high dose radiation to the carotid arteries can lead to vascular disease. Several reports have shown that head and neck radiation using conventional techniques can cause carotid artery stenosis and increase the risk of ischemic stroke [10-13]. Dorresteijn et al assessed 367 patients treated with radiotherapy for head and neck tumors, including 162 patients with larynx carcinomas, and examined the risk of ischemic stroke. The authors found that the relative risk of developing an ischemic stroke in the patients treated for larynx cancer was 5.1, which reached statistical significance [11]. In a more recent study, Smith et al. examined the risk of a cerebrovascular event in patients older than 65 who previously received head and neck radiotherapy. The authors found that the ten-year incidence of cerebrovascular events was 34% in patients treated with radiotherapy alone, compared to 25% and 26% in patients treated with surgery and radiation and surgery alone, respectively [14].

Improving clinical toxicity outcomes by decreasing the dose to normal structures has a precedent in head and neck cancer. Most notably, IMRT is routinely used in locally advanced disease to spare the parotid glands and improve salivary function. More recently, investigators from the University of Michigan have also shown that IMRT can decrease the dose to the pharyngeal constrictor muscles, potentially decreasing rates of long-term dysphagia [15]. In the current dosimetric comparison, we show that IMRT markedly reduces the dose to the carotid arteries compared with conventional radiation without compromising coverage of the PTV. Based on this, it is reasonable to postulate that this reduction in dose will decrease the future rate of radiation related carotid artery disease.

Finally, the concern that IMRT will under-dose the skin and anterior commissure is reasonable but the data in this study suggests that with careful treatment planning, this can be avoided. We have shown that IMRT provides at least equal overall coverage of the entire larynx when compared to 2D techniques, as delineated by our PTV, which includes the anterior portion of the structure. Furthermore, as is the case with non-conformal treatment planning, the routine use of bolus provides an additional safeguard to underdosing anteriorly, though due to the unreliability of dose quantification in the buildup region, the extent of dosimetric improvement in this region is not clear.

It is important to note that our study complements the data of a recent study by Rosenthal et al., which demonstrated that intensity-modulated radiation therapy consistently reduces radiation dose to the carotid arteries, with no compromise in tumor coverage. Furthermore, that study demonstrated that radiation planning and treatment times were similar using conventional techniques versus IMRT [16]. Our study expands on the previous one from a planning standpoint by also including a 3D plan comparison and an analysis of the arytenoid dose, and taken together the conclusion of these two studies is that IMRT can spare normal tissues in early stage laryngeal disease without a decrease in tumor dose, both compared to conventional techniques and 3D conformal therapy.

There are several limitations to the current study. First, we compared techniques in only three patients. In order to maximize the generality of our recommendations, we attempted to select patients with normal anatomy, and patients with both T1 and T2 disease. Second, the impact of organ motion was not assessed in this study. Clearly there is some degree of organ motion when treating the larynx, and the typical boundaries with conventional techniques account for this motion. Whether there is a role for on-board imaging with IMRT will be the subject of a future study. Third, we assessed only one 3D conformal beam arrangement, with the purpose being to compare conventional fields with and without normal tissue optimization/CT planning. The addition of more beams to the 3D conformal plans may offer a better dose distribution, though would likely not have the necessary conformality needed to spare the carotid arteries to the extent of IMRT, as demonstrated in Figure 3d.

Finally, we have shown that IMRT provides dosimetric advantages compared with both 2D and 3D conformal techniques, but the clinical significance of such dose reduction is not known. One criticism of our findings may be that it is no surprise that when comparing target and normal tissue dosimetry between two dimensional and conformal techniques (3D and IMRT), the latter methods provide a more optimal dose distribution from a physics standpoint. However, we believe that this study is important because it has shown that when using "appropriate" tumor margins for this disease, IMRT can provide potential long-term clinical advantages even in the context of the relatively small fields and the unique anatomic relationships that are present in the treatment volumes for early-stage glottic laryngeal cancer. We also understand that the utility of IMRT in this disease, and any recommendations that can be drawn from this study, will depend on defining the adequate target volume. Extrapolating from other head and neck sites in which IMRT is utilized and in which excellent rates of local control have been achieved, we believe that CTV to PTV margins of 0.5 - 1.0 cm are reasonable for glottic laryngeal cancer. With this underlying assumption, our data supports the recommendation that IMRT should be strongly considered for this cohort of patients, Based on the dosimetric findings in this study, a reasonable cost-effective treatment paradigm would be: 1) A 2D "tight" clinical plan, a 3D conformal plan with a 0.5 cm margin, or, ideally, an IMRT plan with arytenoid sparing in the case of anteriorly located T1N0 disease, and 2) IMRT in the case of posteriorly located or bulky T2 lesions, where this technique could be used to spare the carotid arteries better than 2D or 3D conformal plans. Perhaps the most important conclusion that can be drawn from this study is that regardless of what is determined to be the appropriate margin in delineating the CTV (and thus the PTV) for early laryngeal cancer, IMRT maximizes the freedom of the clinician to choose a margin that is most appropriate for them. Or put another way, the more confident a clinician is about the PTV, the more of an advantage IMRT offers over other techniques.

## Competing interests

The authors declare that they have no competing interests.

## Authors' contributions

DG - Primary author of manuscript and revisions. OC - Contributed to writing of manuscript and concept. JM - Performed physics plans and assisted with manuscript. NL - Concept of paper, contributed in writing manuscript and all revisions. All authors read and approved the final manuscript.
